# Mechanisms Involved in Cellulose Depolymerization by Treatment with Energetic Beams

**DOI:** 10.3390/polym18182220

**Published:** 2026-09-11

**Authors:** Miran Mozetič

**Affiliations:** Department of Surface Engineering, Jozef Stefan Institute, Jamova cesta 39, 1000 Ljubljana, Slovenia; miran.mozetic@ijs.si

**Keywords:** cellulose, bioethanol, electron beam, depolymerization, efficiency

## Abstract

Depolymerized cellulose is a precursor to bioethanol, an attractive solution for the green economy. A method for cellulose depolymerization is treatment with energetic particles that break the bonds between glucose units. The scientific literature on depolymerization by energetic electrons is reviewed and analyzed. If all electron energy is spent on complete depolymerization to fermentable sugars, the theoretical dose is estimated to be about 1000 kGy. The experimental results, however, indicate rather poor energy efficiency, which is attributable to several factors. Regardless of the cellulose source, experimental setup, and electron energy, the results reported by various teams fit a general curve. The degree of polymerization drops by an order of magnitude after receiving the dose of about 70 kGy. The general curve is explained by secondary effects, including the generation of X-rays with favorable penetration depth, which produce rather low-energy electrons and photons useful for scission of the β(1→4)-glycosidic bonds. The solubility of depolymerization products in water increases with dose and reaches 10% at a few 100 kGy. Depolymerization to simple oligosaccharides or monosaccharides is not feasible due to inadequate selectivity and is energetically too demanding.

## 1. Introduction

Fossil fuel supplies are limited and unevenly distributed, and timely delivery to users could be a subject of political instability. While electrification may replace standard fuels for vehicles and other uses, the costs of electricity storage devices remain a serious limitation. Alternatives include green hydrogen and bio-based fuels [1,2,3]. Among the latter, bioethanol has been added to standard fuels for years: it is now produced on a large scale from sugars and starch, but production remains limited despite significant increases in crop yields enabled by modern agricultural methods. An ideal source of bioethanol would be organic waste rich in cellulose and other polysaccharides. In fact, the synthesis of bioethanol from lignocellulosic biomass is a hot research topic, with numerous articles published in recent years. The scientific backgrounds and technological challenges are explained in several review articles, including [4,5,6,7,8,9,10,11]. The lignocellulosic mass cannot be transformed directly into bioethanol because it is not fermentable. It should be broken down to fermentable sugars, and the common method is enzymatic depolymerization. The sugars are then converted into ethanol through fermentation [12], and the net effect is that a glucose molecule is converted into two ethanol molecules and two carbon dioxide molecules.

**Figure 1:**
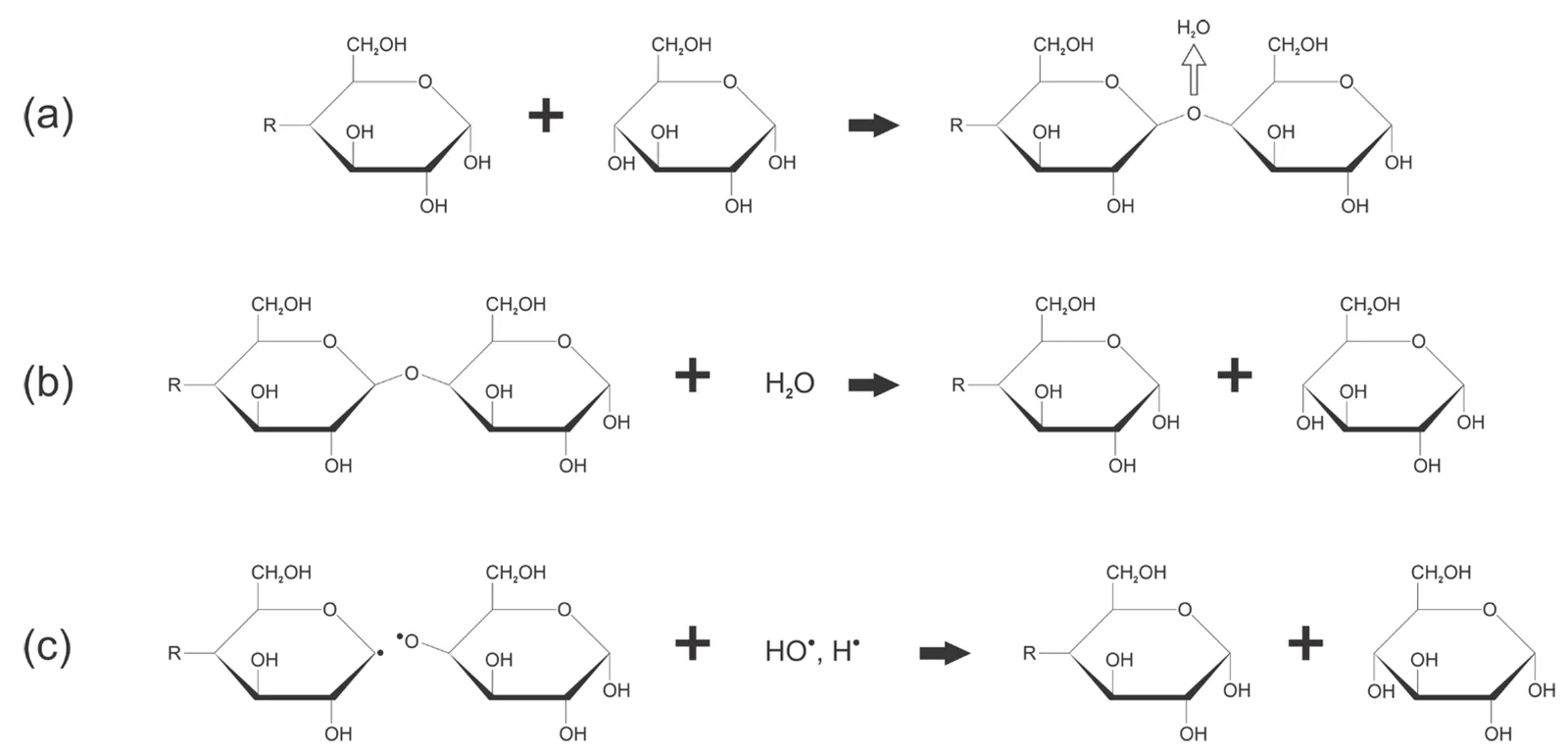
Illustration of the condensation reaction (**a**), depolymerization to yield glucose (**b**), and depolymerization using electron-beam irradiation (**c**).

Cellulose is the most abundant natural polymer and consists of large chains of glucose units. Plants synthesize cellulose by a condensation reaction, forming β(1→4) linked D-glucose units, as illustrated in Figure 1a.

The opposite reaction, i.e., the release of a glucose molecule from the cellulose chain, could occur if the β(1→4) bond is broken in the presence of a water molecule (reaction in Figure 1b). However, such an opposite reaction does not occur for numerous reasons [13], the most trivial of which is the reaction barrier and endothermic character of the reaction (b), opposite to the condensation (a). Heating wet cellulose will cause polymer degradation, producing numerous products rather than pure glucose. A bio-inspired solution is the application of enzymes. An enzyme can break the β(1→4) bond, but the process is slow and inefficient due to several factors [14]. The cost, however, is prohibitively high, so the production of fermentable material from cellulose remains the subject of extensive research. The key step (reaction in Figure 1b) is depolymerization, i.e., breaking the cellulose chain to fermentable water-soluble products, preferably glucose. Cellulose is insoluble in water, but its depolymerization products, such as sugars, are highly soluble.

The number of glucose units in a cellulose molecule is often expressed as the degree of polymerization (DP), defined as the average number of glucose units per cellulose molecule. The degree of polymerization of natural cellulose is roughly between about 1000 and 10,000 [15]. The cellulose molecules in a macroscopic sample exhibit different degrees of polymerization, resulting in a rather broad distribution. The degree of polymerization has been determined by various techniques. The most common are viscosity measurement and gel permeation chromatography. Other techniques have been reported, including the osmotic pressure method, ultracentrifugation, end-group chemical analysis, light scattering, and nuclear magnetic resonance spectroscopy. The basic principles of these methods have been described in [16].

**Figure 2:**
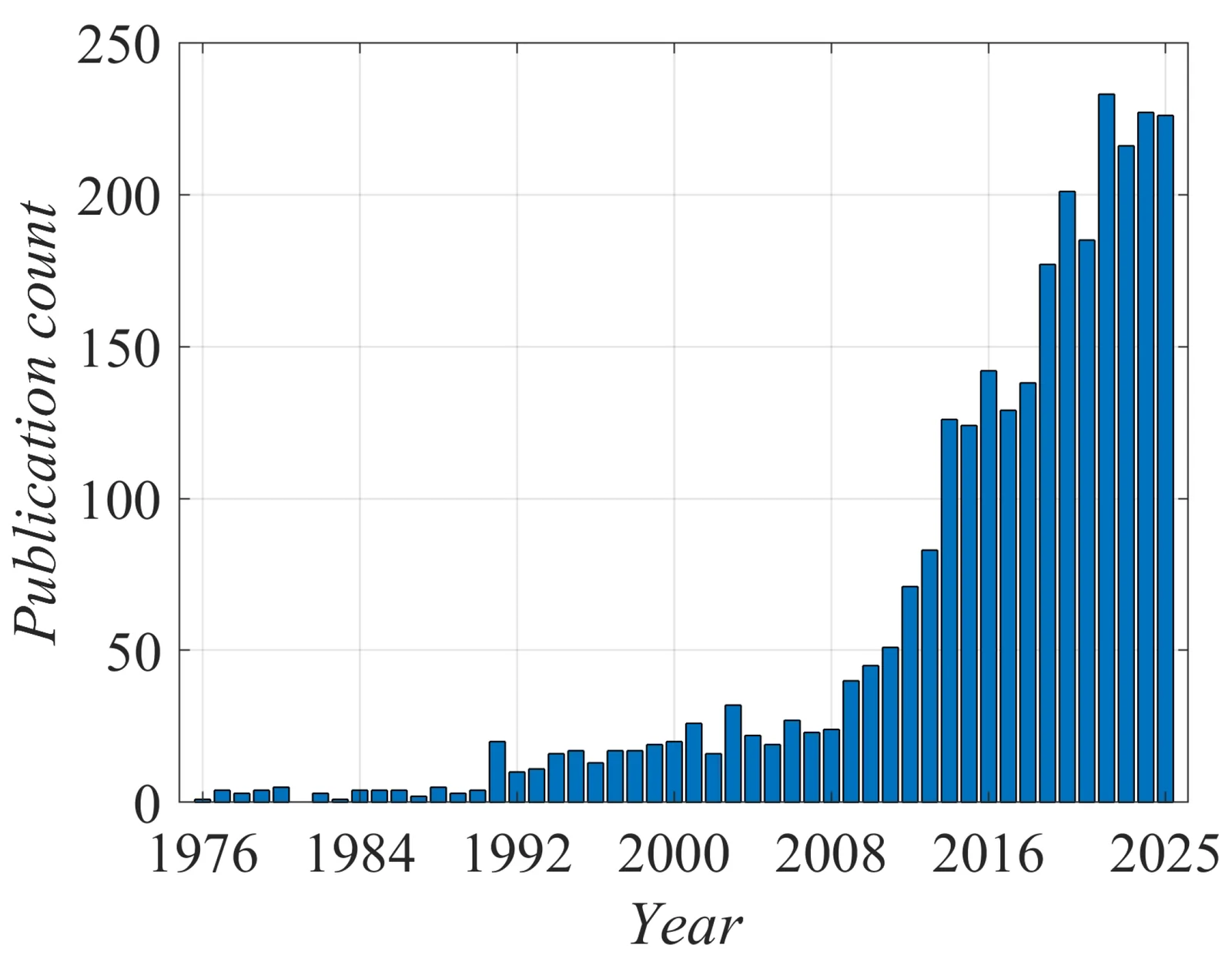
Number of publications on cellulose depolymerization.

Cellulose depolymerization has been probed by various techniques. Figure 2 shows the number of scientific papers recorded in the Web of Science. The topic was only briefly addressed until 2008, after which a significant increase was observed. The number of publications increased tenfold in the next 10 years. Apart from the bioinspired procedure (milling and purification of the suspension, and the application of enzymes to depolymerize cellulose), recent approaches include photochemical degradation [17,18], hydrothermal catalytic hydrolysis [19], molten salt hydrates [20], and subcritical water hydrolysis at very high pressures [21]. While some methods appear scalable and potentially inexpensive (like algal photobioreactors and genetically modified plants and/or microbes), tremendous scientific effort is needed to develop a method that will be used in the future as a key step in the synthesis of bioethanol from cellulose, preferably from plant wastes such as wood waste, straw, and similar materials.

The application of electron beams to modify lignocellulosic biomass has been considered a method for biomass pretreatment [22,23,24,25], which not only induces structural modifications but also increases the cellulose content [26] and has been found useful for disinfection [26].

Given the simplicity of the reaction shown in Figure 1b, one possible approach is to break the β(1→4) bonds by irradiating cellulose with particles of appropriate energy. A standard method for rather selective bond cleavage is the application of non-equilibrium gaseous plasma [27]. Reactive plasma species are energetic enough to break chemical bonds in organic matter. Furthermore, water is likely to dissociate into H and OH radicals under plasma conditions. The dangling bonds on the cellulose surface are likely to interact with H and OH radicals, so the opposite reaction as Figure 1a seems feasible. The reaction is illustrated in Figure 1c. In fact, some authors reported a high degree of depolymerization and high production of water-soluble fragments, including sugars [27]. Plasma treatment thus seems feasible, but it has a major drawback: plasma species are unlikely to diffuse into cellulose, so the reactions are limited to the very thin surface film. Hence, there is a need to create a similar effect in the bulk cellulose. It is impossible to sustain plasma within cellulose, so alternative methods should be applied.

## 2. Cellulose Depolymerization by Irradiation with an Electron Beam

Several energetic particles can penetrate cellulose (and other organic materials). Energetic particles are either positively charged ions, electrons, or photons. Positively charged ions are likely to cause a collision cascade in the surface film through elastic collisions with atoms in the solid material, thereby significantly heating it, and are therefore not applicable for cellulose depolymerization. Furthermore, the penetration depth of energetic ions is well below 1 µm even for 100 keV ions [28]. Energetic photons, such as gamma rays, will penetrate deeply into cellulose [29], but the available fluxes are by far too low to be considered an option. Energetic electrons are readily produced by heating a cathode and accelerating them in a vacuum chamber. The vacuum chamber could be separated from the cellulose by a thin, almost transparent membrane, thereby making electron treatment feasible. Not surprisingly, it was one of the first techniques for cellulose depolymerization to be published in the scientific literature. The aim of this paper is to analyze the literature, draw correlations, and explain the effects of energetic beams on the cellulose chemistry.

### 2.1. Brief Literature Survey

Electron beam irradiation of cellulose has been a research topic for decades. The early papers report various treatments that were found to be beneficial or detrimental for specific purposes [30,31], but the authors did not report depolymerization as a function of dose. The topic remains a scientific interest [32,33,34,35,36], but most authors do not quantify depolymerization.

**Figure 3:**
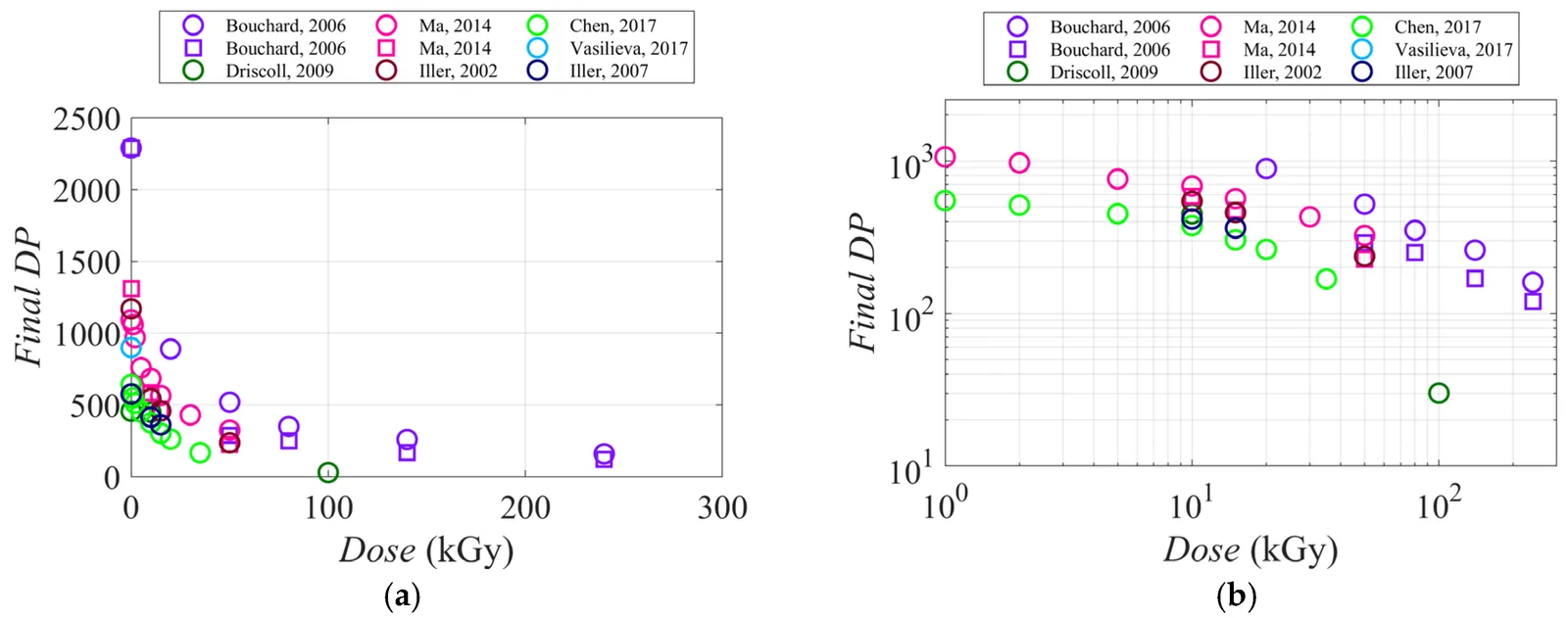
The degree of polymerization of cellulose samples versus the dose of energetic electrons. (**a**)—linear scale, and (**b**)—logarithmic scale on the y-axis [37,38,39,40,41,44,45].

One of the first quantitative reports on the depolymerization of cellulose by electron-beam irradiation was provided by Bouchard et al. [37]. The aim was to evaluate the feasibility of inactivating bacterial spores without significantly damaging the paper through electron-beam treatment. The authors used electrons at energies of 4.5 and 10 MeV and measured the degree of polymerization as a function of the energy dose. Their results are plotted in Figure 3, together with results reported by other authors.

Iller et al. [38] treated Alicell, Borregaard, Ketchikan, Kraft softwood, and Kraft hardwood pulps with 10 MeV electrons. After receiving the dose of 50 kGy, the fraction of cellulose with a polymerization degree over 550 dropped for an order of magnitude or more for most samples, while the fraction of cellulose below 200 decreased moderately, about 2–3 times. In another experiment [39], the same team also treated cellulose pulps with 10 MeV electrons and studied structural and physicochemical properties. The electron-beam treatment did not affect the production of carboxymethylcellulose from pinewood cellulose pulps, but it was found promising for the synthesis of cellulose carbamate.

Driscoll et al. [40] used a powerful (90 kW) source to treat microcrystalline cellulose with 1 MeV electrons. After receiving the dose as large as 1000 Gy, the relative crystallinity dropped by a factor of 2, and the surface area of the microcrystalline cellulose increased by about 20%, but the average weight of the cellulose molecules dropped by a factor of 40, indicating that the depolymerization was the major result of the irradiation.

Vasilieva et al. [41] used moderate-energy electrons for treating microcrystalline cellulose powder. They used a reactor in which an extremely nonequilibrium plasma was sustained by an electron beam [42]. The energy of primary electrons was about 30 keV. The depolymerization was studied as a function of treatment time, and the dose could be estimated by considering another report by the same author [43]. Vasilieva et al. observed rapid depolymerization, and the concentration of water-soluble products in distilled water was as large as 77% after treating the powder for 10 min. As already mentioned, untreated cellulose is insoluble in water. The treated cellulose powder was 100% soluble in 5% NaOH.

Ma et al. [44] irradiated bamboo chips of thickness 5 mm with an electron beam from an electron accelerator operating at a power of 15 kW. Rapid depolymerization was observed, but the crystallinity did not change significantly. The same team also treated bamboo pulp using the same electron-beam source and observed a gradual decrease in degree of polymerization, with Fock reactivity approaching 100% and negligible cellulose loss at moderate doses [45].

**Figure 4:**
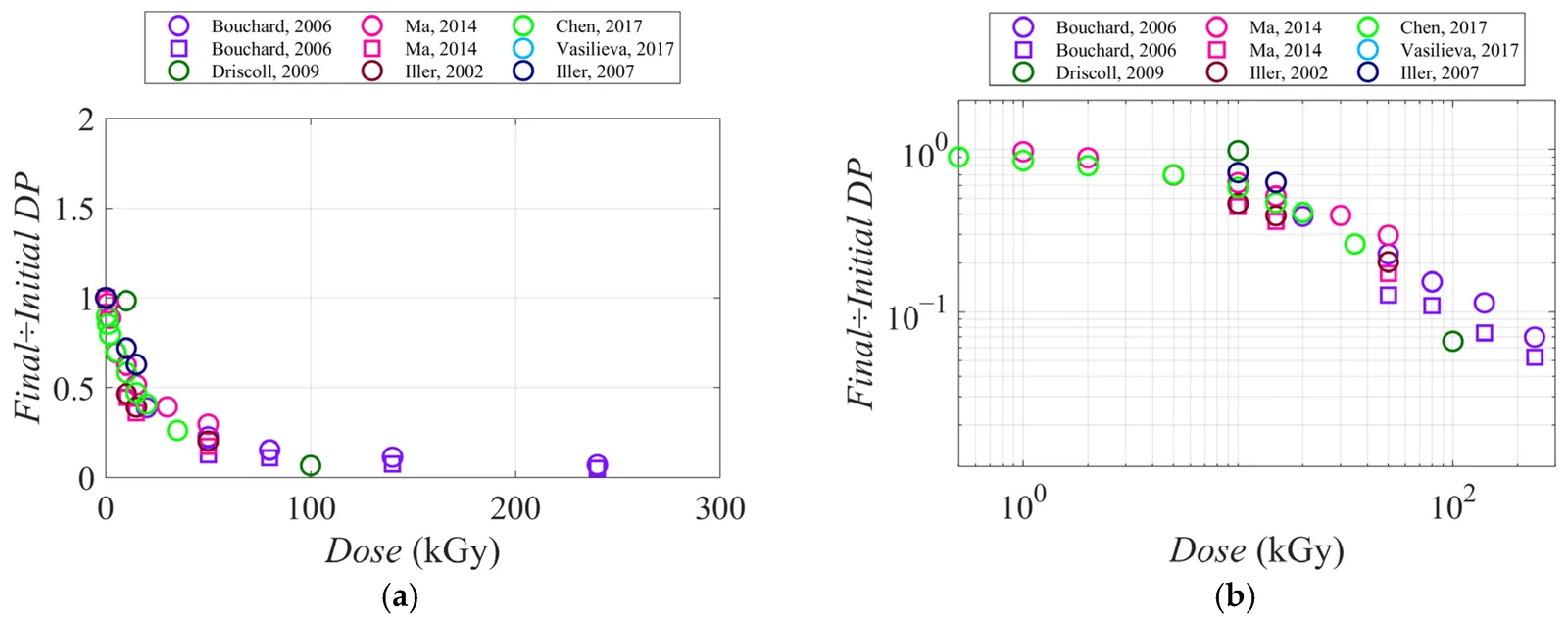
The normalized degree of polymerization of cellulose samples versus the dose of energetic electrons. (**a**)—linear scale, and (**b**)—logarithmic scale on the y-axis [37,38,39,40,41,44,45].

Figure 3a shows the degree of polymerization (DP) of cellulose samples versus the energy dose of energetic electrons. The result is impressive—the DP decreases quickly up to a dose of a few 10 kGy, and more gradually thereafter. Most authors focused on rather small doses, so the measured points in Figure 3a are concentrated near the origin. To better illustrate the measured values, the same points are shown on a logarithmic scale in Figure 3b. Figure 3 clearly shows a general trend, but some measured points deviate significantly from the general curve. Such deviation is at least partially due to the differences in the original DP. Therefore, the normalized DP, i.e., the degree of polymerization after the electron-beam treatment divided by the initial DP, is plotted in Figure 4. Despite some scattering, Figure 4 clearly shows that all measured points lie on the same curve.

**Figure 5:**
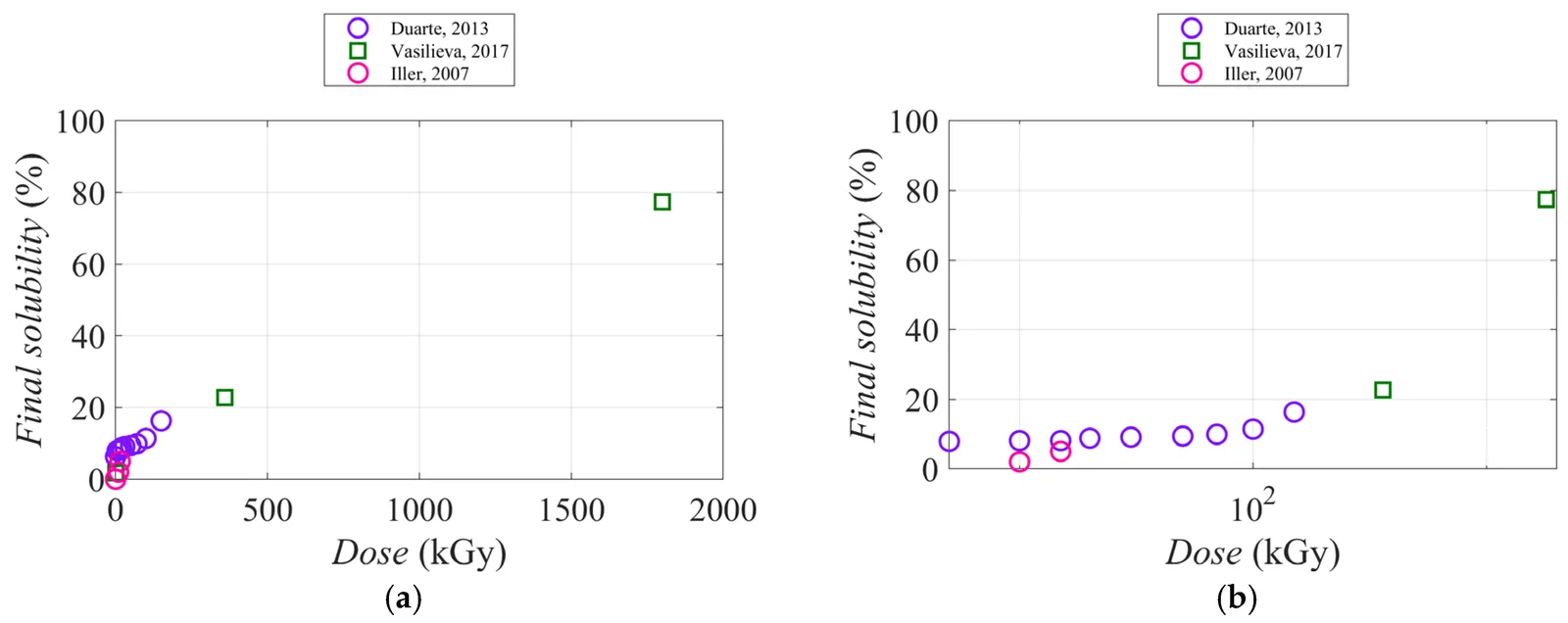
The solubility of cellulose samples treated with an electron beam versus the energy dose, (**a**) in a linear x-axis scale, and (**b**) in a logarithmic x-axis scale [39,41,46].

The solubility of partially depolymerized cellulose in water is an important parameter indicating the formation of water-soluble products. Namely, cellulose is insoluble, and many sugars are highly soluble in water. Unfortunately, the solubility of electron-beam-treated cellulose samples in water has been reported in only three articles [38,41,46]. The solubility of cellulose samples in water after electron-beam treatment is plotted in Figure 5. Figure 5a shows the solubility on a linear scale on the x-axis, and Figure 5b shows it on a logarithmic scale. All authors whose results are plotted in Figure 5 observed a significant increase in solubility, which increased monotonically with dose. Duarte et al. [46] treated sugarcane bagasse (the pulpy fibrous residue that remains after crushing sugarcane stalks to extract their juice) with 1.5 MeV electrons but did not report the degree of polymerization; therefore, the article is not mentioned in the literature review above. The team also probed solubility after hydrothermal treatment of the irradiated samples, and reported a significant increase. The hydrothermal treatment was performed in pure water at a temperature of 180 °C. The main monosaccharide reported by Duarte et al. [46] after hydrothermal treatment of electron-beam-treated bagasse was xylose, which is a monomer obtained after the depolymerization of hemicelluloses. Xylose fermentability is still a subject of scientific research [47].

### 2.2. Interaction of Fast Electrons with Cellulose

The results summarized in Figure 4 are worth discussing. Namely, different teams used different electron beam sources, and the electron energy spans over several orders of magnitude. For example, Vasilieva et al. [41] used the electron beam of electron energy as low as about 30 keV, while Bouchard et al. [37] and Iller et al. [38] used beams with the electron energy as high as 10 MeV, i.e., 300 times larger. Still, the results fit the same curve. Furthermore, different teams used various sources of cellulose, so the general curve observed in Figure 4 is rather surprising and needs clarification.

A dose of 1 Gray (Gy) is equal to the absorption of 1 Joule of radiation energy per 1 kg of matter, so it is a measure of the absorbed energy. The number of electrons impinging on the sample that have received the same energy dose decreases with increasing electron energy. The number of electrons (*n*) impinging on a sample of mass (*m*) is

**Figure 6:**
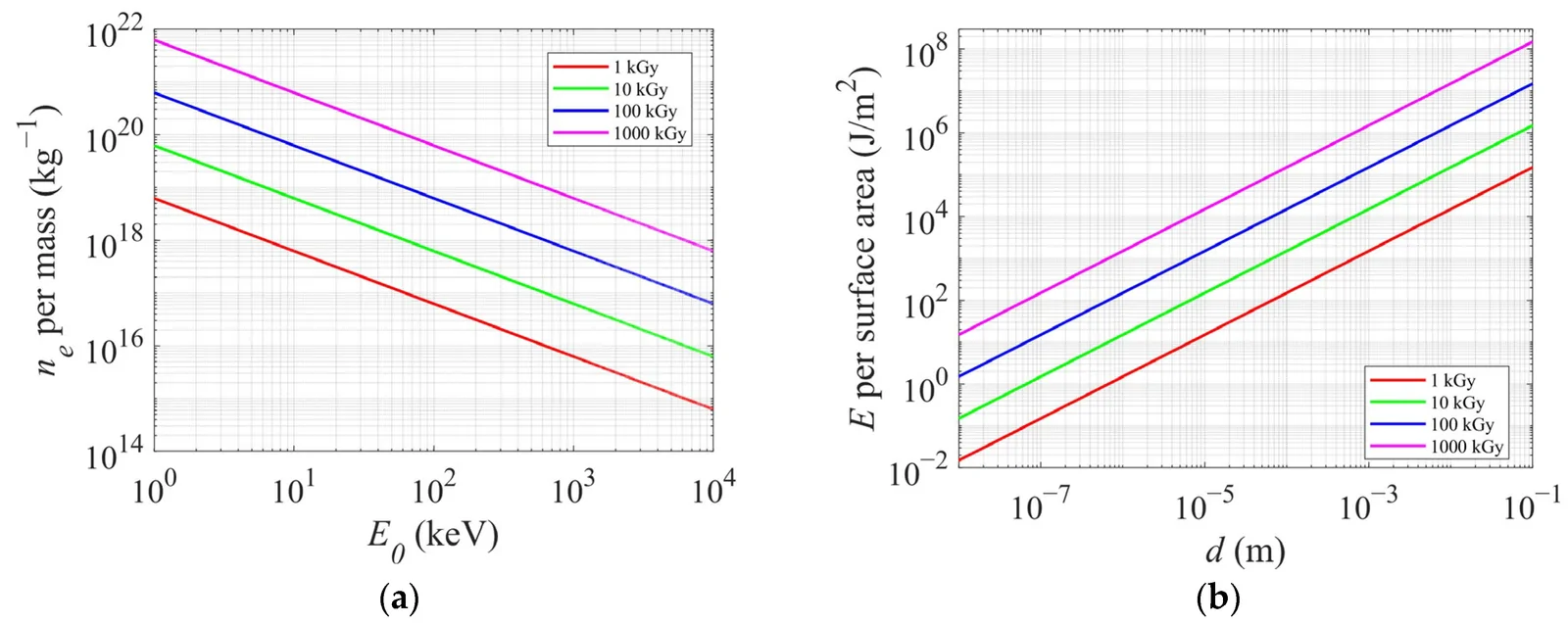
(**a**) The normalized number of electrons impinging on the cellulose sample versus the energy of impinging electrons. (**b**) The energy dissipated on the unit surface of cellulose samples versus the sample thickness.

*n*/*m* = *dose*/*E*_0_, (1)

where *E*_0_ is the energy of an impinging electron. Figure 6a represents the normalized number of electrons (number divided by the mass of the treated object) versus the kinetic energy of impinging electrons. The dose is the parameter. The number of electrons is rather low. For example, at the electron energy of 1 MeV and the dose of 10 kGy, the number of electrons impinged on a cellulose sample is roughly 10^17^ kg^−1^. The number of cellulose molecules with a degree of polymerization of 1000 in 1 kg of cellulose is

*N*/*m* = *m*_cell_^−1^ = 3 × 10^21^ kg^−1^. (2)

Here, *m*_cell_ is the mass of a cellulose molecule with DP 1000 (1.8 × 10^5^ Da = 3 × 10^−22^ kg). The number of β(1→4) linked D-glucose units in a cellulose molecule with DP 1000 is obviously 1000 times larger than the number of cellulose molecules, i.e., roughly 10^24^ kg^−1^. The number of electrons impinging on the cellulose sample is, thus, by far too low to cause significant depolymerization. Considering Figure 4, it is obvious that a single electron breaks many bonds upon impinging on a cellulose sample.

The energy dissipated on the surface of cellulose samples with area S upon electron irradiation will depend not only on the absorbed dose but also on the sample thickness. The energy dissipated per unit surface area is

*E*/*S* = *dose*
*ρ*
*d* = *E*_*0*_
*n*/*S*, (3)

where *ρ* is the cellulose density (roughly 1.5 × 10^3^ kg/m^3^), and *d* is the sample thickness. The energy calculated using Equation (3) is plotted versus the thickness of the cellulose sample in Figure 6b. It is clear that the depolymerization efficiency should also depend on the thickness of the irradiated samples, not only the dose. The articles whose results are summarized in Figure 4 and Figure 5 do not report sample thickness (except [44]), so it is not possible to plot DP versus thickness. In any case, the thickness dependence shown in Figure 6b should be taken into account when interpreting the general trend revealed by those figures, as it is not expected.

Electrons with reasonable energy will not penetrate deeply into the cellulose samples. In a first approximation, the penetration depth of electrons at rather low energies (roughly between 1 and 100 keV) is calculated from the Kanaya–Okayama equation [48]:

(4) $$R_{KO}=\frac{0.0276\;A\;E_{0}^{1.67}}{Z^{0.89}\rho }.$$

Here, the penetration depth is in µm, *A* is the average atomic weight of cellulose (about 7.5 kg/kmol), and *Z* is the average atomic number of cellulose (about 4.1). The cellulose density should be in g/cm^3^, and the kinetic energy of electrons should be in keV. Equation (4) is empirical, i.e., the best fit obtained from experimental results.

Equation (4) is replaced with another empirical formula for the case of more energetic electrons. If *E*_0_ is between 0.01 and 10 MeV, the approximation provided by Katz and Penfold [49] applies:

(5) $$R=412E_{0}^{1.265-0.0954\ln E_{0}}.$$

Here, *E*_0_ is in MeV, and *R* is in cm.

**Figure 7:**
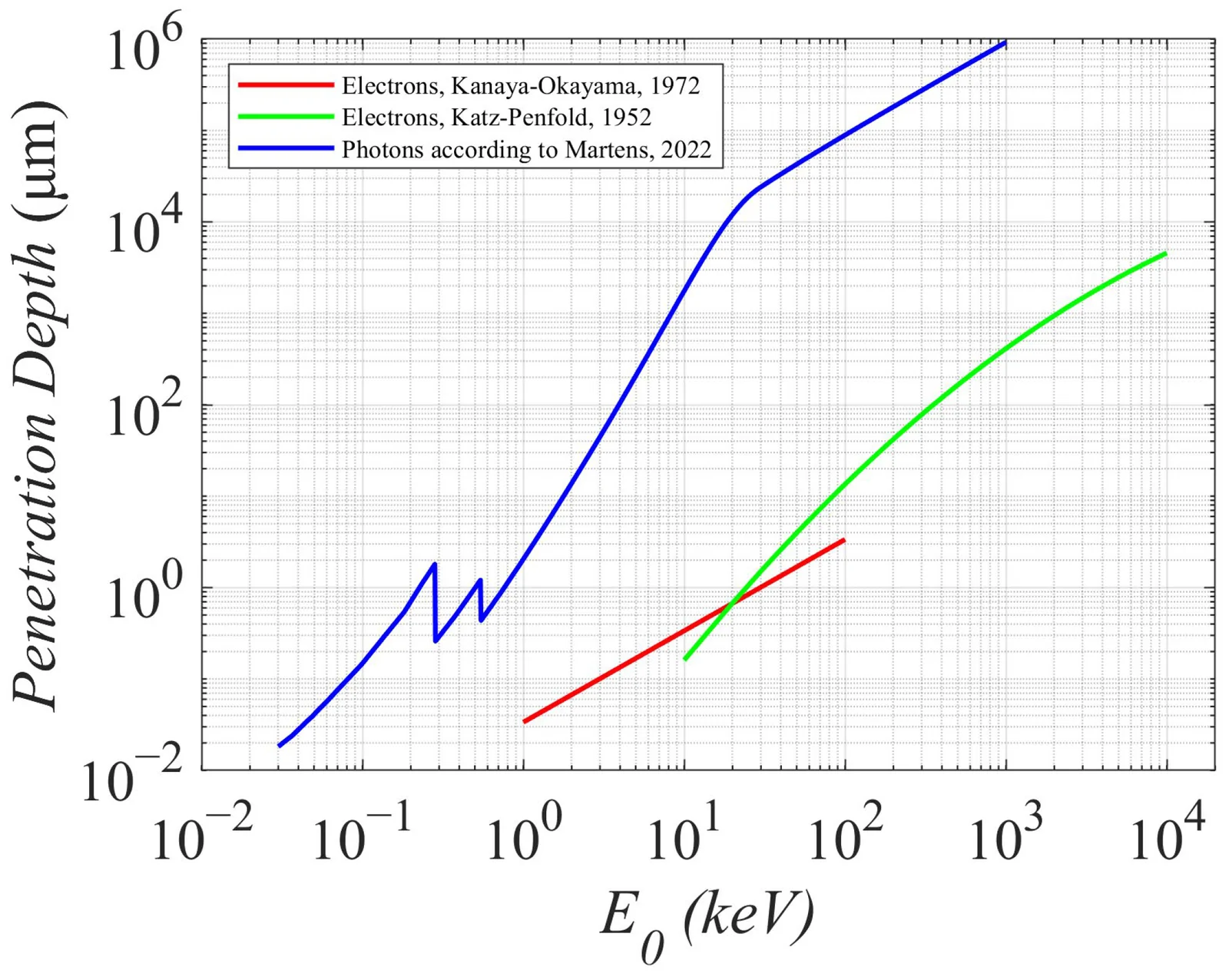
The penetration depth of electrons and photons in cellulose samples as derived from Equations (4) and (5), and [48,49,51].

The penetration depths for electrons in the range of energies between 1 keV and 10 MeV are plotted in Figure 7. The penetration depth is only 30 nm for electrons with a kinetic energy of 1 keV, and about 4 mm for 10 MeV electrons. The results represent only a rough estimate. Details on penetration depths and energy absorption upon electron irradiation of cellulose at electron energies of 150–3000 keV were provided by Li et al. [50]. In any case, the penetration depth of primary electrons is by far too low to make the electron-beam treatment a feasible method for depolymerization of bulk cellulose samples.

Taking into account the upper considerations, the rather slow electrons will modify only a very thin surface film on cellulose samples, whereas very thin cellulose samples are likely to be partially transparent to energetic electrons. The large variation in penetration depth with the kinetic energy of impinging electrons further complicates the explanation of the general curves shown in Figure 3, Figure 4 and Figure 5.

Taking into account simple calculations whose results are presented in Figure 6 and Figure 7, and the results on depolymerization and water solubility summarized in Figure 4 and Figure 5, it is evident that the electrons used for the irradiation of cellulose materials are not the major reactants. Namely, if they were, the depolymerization should be marginal even after a very high irradiation dose (taking into account the ratio of primary electrons to β(1→4) bonds), and the results would depend significantly on sample thickness. For example, cellulose in the form of a fine powder or thin paper will be semi-transparent to fast electrons, so most electrons will miss the target, and only a very thin surface film of thick samples treated with relatively low-energy electrons would be modified.

### 2.3. Electron Energy Loss Mechanisms

The fast electrons are scattered as they propagate through a solid material. The scattering will cause numerous effects [52], and the most relevant are mentioned below. Incident electrons will collide with electrons in solid (or liquid) materials, displacing them from their original positions. The energy of an incident electron scattered on a bonded electron is shared between both electrons, and a small fraction is spent on the liberation of the bonded electron. The result is the formation of many free electrons with various energies, which are commonly called secondary electrons. The distribution of secondary electrons is a continuum, except for a small amount of Auger electrons, which are stretching from a broad continuum of secondary electrons. Considering Figure 7, the penetration depth of secondary electrons is lower than that of the primary electrons.

**Figure 8:**
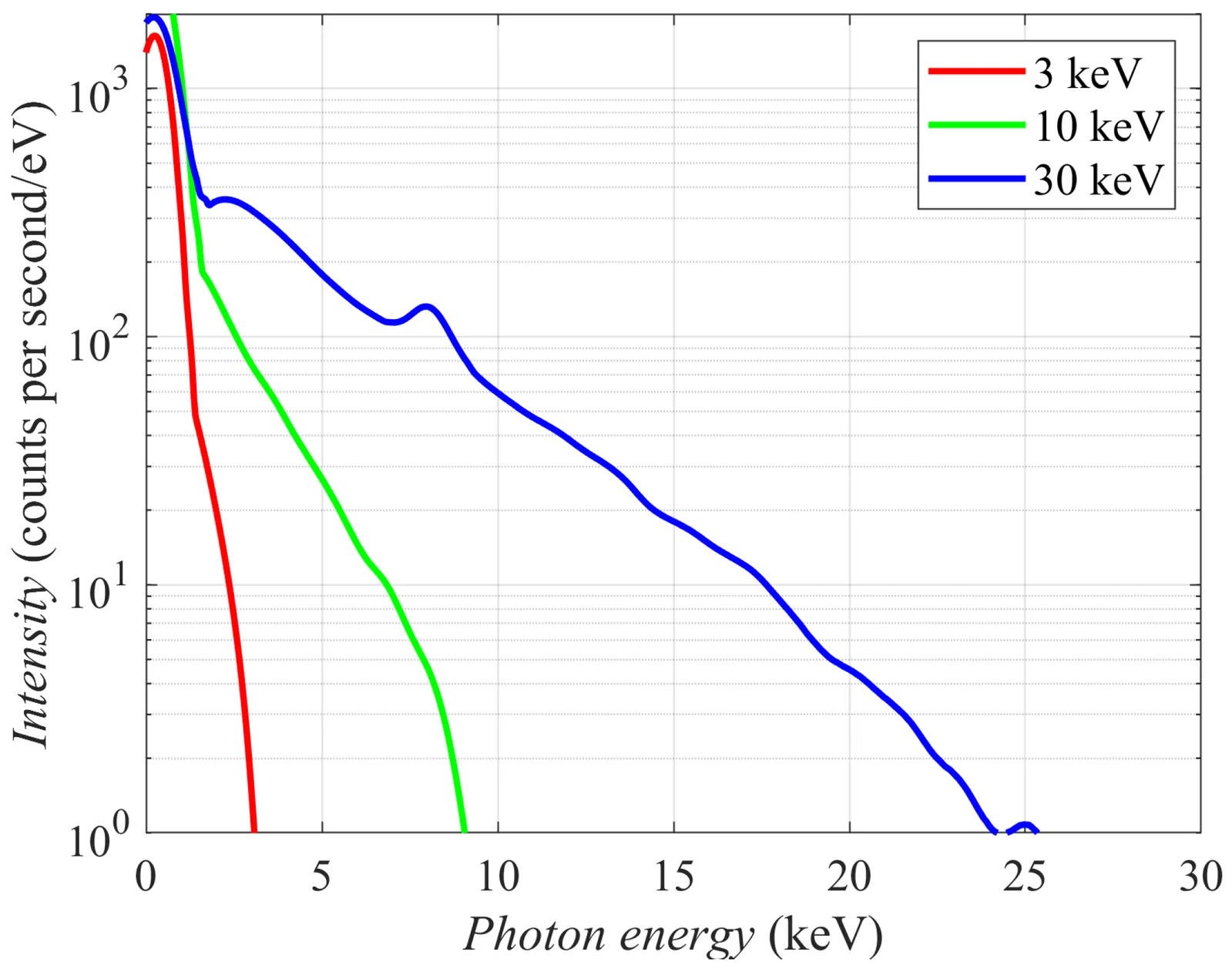
Photon spectra of cellulose irradiated with monochromatic electrons of the energies 3, 10, and 30 keV.

Both primary and secondary electrons also lose energy by radiation, i.e., by emitting photons. The major channel for the radiative loss of the fast electrons’ energy is bremsstrahlung. The electrons passing a high electric field (like an atom nucleus) radiate photons, and the loss of electron energy is equal to the energy of emitted photons. The photon radiation intensity depends on the electric field strength, i.e., the minimal distance between the nucleus and the electron trajectory. The distance varies among impinging electrons, so the spectrum is a continuum. Figure 8 shows the spectra of photons produced by an electron beam upon impinging on a cellulose sample. The majority of radiation forms a continuum, and the peak stretching from the continuum at relatively low photon energy arises from photons emitted when a hole in an atom’s inner shell (which has been created by an inelastic collision of a primary electron with an electron from an inner shell) is filled by another electron (characteristic radiation). The radiation intensity increases significantly with increasing primary electron energy, and the distribution broadens, as shown in Figure 8.

The primary electrons thus create photons in a broad range of energies. The photons will be emitted in all directions, and those directed towards the bulk cellulose will be absorbed in the bulk. The penetration depth of energetic photons in cellulose (and many other materials) is much larger than the penetration depth of electrons with the same energy. It can be calculated using a penetration depth calculator from TU Wien, Wien, Austria [53]. The estimated penetration depth from this approximation is shown in Figure 7 with a blue curve. The penetration depth of photons is much larger than that of electrons of the same energy, roughly 4 orders of magnitude. That is what makes X-rays suitable for medical screening of various tissues. The suitable X-ray energy is between approximately 10 and 100 keV, depending on absorption in different tissues [54]. Here, it is worth noting that the penetration depth of photons with energies below the photoionization thresholds of the inner electron shells in carbon and oxygen is not a smooth curve; sharp drops are observed as the photon energy approaches the threshold [55].

Photons will also be absorbed by cellulose. There are two major channels—photoionization (which prevails at relatively low photon energies (up to about 30 keV)), and Compton scattering. In the first case, a photon is absorbed during photoionization, releasing an electron. Compton scattering is the inelastic scattering of a high-energy photon (such as X-rays) by a loosely bound electron. During the collision, the photon transfers some of its energy to the electron, causing the electron to recoil and the photon to deflect with reduced energy and an increased wavelength.

**Figure 9:**
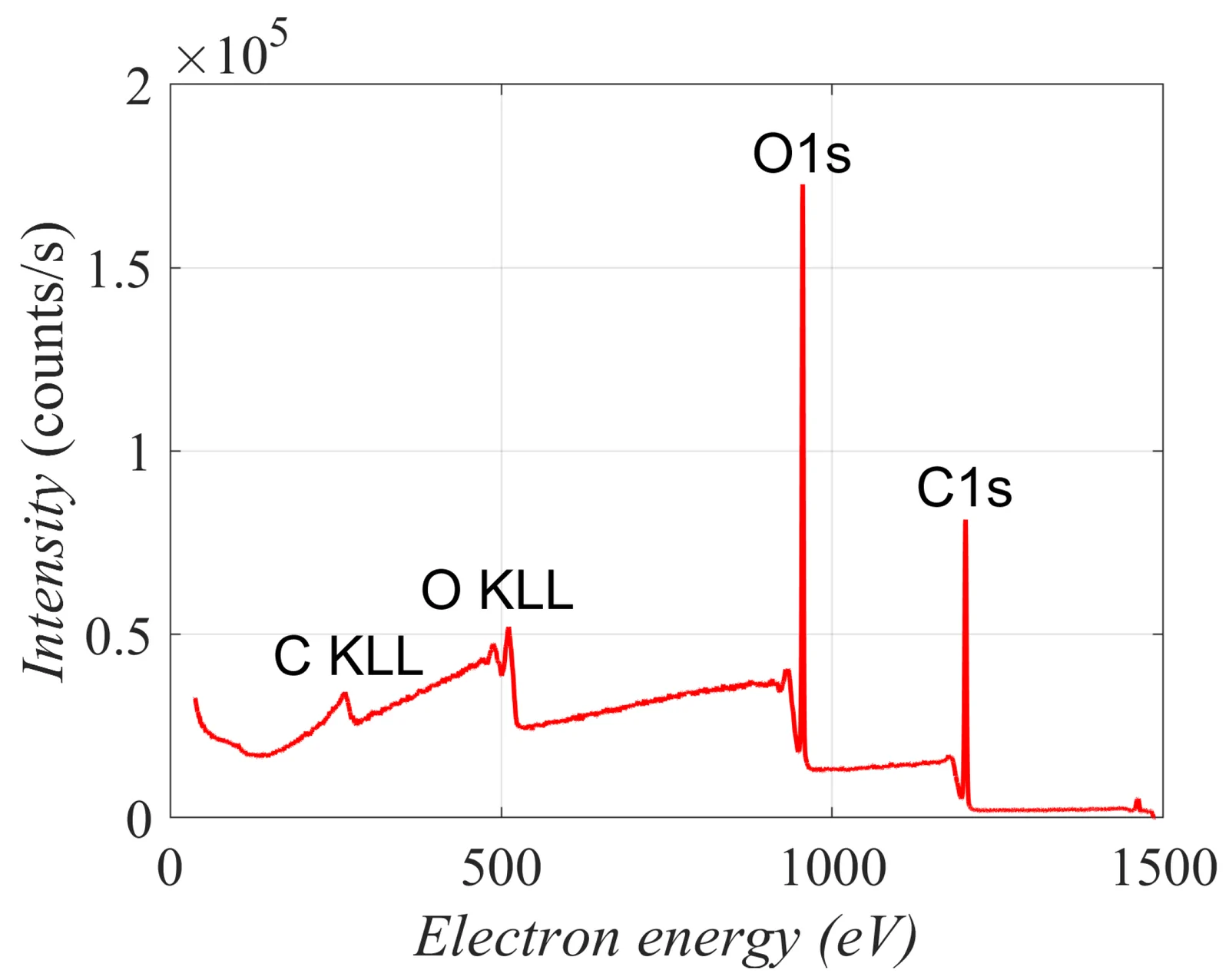
A spectrum of electrons emitted from cellulose upon irradiation with photons of energy 1486.6 eV.

Figure 9 shows a spectrum of emitted electrons upon irradiation of cellulose with monochromatic photons at an energy of about 1.5 keV. The spectrum reveals a step-like continuum with steps occurring at the threshold photon energy for photoionization of inner shells (1s) of carbon and oxygen atoms. The binding energies of the 1s shells are about 285 eV and 530 eV for carbon and oxygen, respectively. The sharp peaks observed at the electron energy of about 950 and 1200 eV correspond to photoelectrons that have not suffered a collision between the emission site and the electron detector. The continuum corresponds to the secondary electrons (i.e., those that have been scattered). The photon absorption thus produces numerous, relatively slow electrons near the absorption site in the bulk cellulose.

### 2.4. The Qualitative Illustration of the Cellulose Depolymerization by Electron Beams

The upper explanations could be summarized as follows. Primary electrons with a limited penetration depth will cause an avalanche of slower electrons and photons. Some primary electrons will also be backscattered and thus lost for the modification of a cellulose sample. The slower electrons (secondary electrons) will not penetrate deeply but will also produce energetic photons, as evidenced in Figure 8. The photons will penetrate deep into the cellulose sample, where they will release rather slow electrons, as demonstrated in Figure 9. According to Figure 7, the penetration depth of energetic photons is orders of magnitude larger than the penetration depth of electrons with the same energy. The photons will thus serve as a vector for delivering low- or moderate-energy free electrons deep into the cellulose sample. These electrons will, in turn, generate relatively low-energy photons as evidenced in Figure 8. The final effect of the exchange of energy between the electrons and the photons will be numerous slow electrons and photons of relatively low energy formed deep in the cellulose sample.

Electrons with energies around 10 eV will cause bond scission deep inside the cellulose samples, but the effect is yet to be quantified. The same applies to photons of the energy range around 10 eV. As early as 1969, Desai et al. reported that chain scission is the primary process yielding glucose and oligosaccharides upon ultraviolet irradiation [56]. Gas chromatography of the volatile products revealed additional degradation products, including acetaldehyde, propionaldehyde, methyl formate, acetone, methanol, ethanol, methane, and ethane. The irradiation with the UV photons is, thus, not very selective, so not all photons are used for scission of the β(1→4) bond. The bond scission is particularly intense for photons in the vacuum ultraviolet (VUV) range, i.e., between about 100 and 200 nm, corresponding to photon energies between 6 and 12 eV. In their classical paper, Fouchier et al. [57] studied the interaction of VUV radiation with several polymers and found highly efficient bond scission in all cases. The scission of the C–O–C bond was clearly distinguished, but other bonds were affected, too. This is further evidence of the non-selectivity of photon-induced bond scission at energies around 10 eV.

The bond scission induced by the interaction between relatively slow electrons and photons (with energies around 10 eV) with cellulose has been demonstrated in the scientific literature [58]. Photons of moderate energy (deep UV and VUV) are thus likely to break bonds, thereby modifying the cellulose structure and composition [59,60,61]. Still, the effect does not necessarily lead to cellulose depolymerization. Namely, the displaced electron could assume the same bonding site as before displacement, so the reaction is at least partially reversible.

According to the reverse effect of condensation (Figure 1c), the dangling bond formed by scission of the β(1→4) bonds should be occupied with OH radicals to form a glucose molecule. The reaction illustrated in Figure 1c will occur if a water molecule is present near the position of the β(1→4) bond. Cellulose is never perfectly dry, so water is present in all practical cases. The formation of glucose after scission of the β(1→4) bond will be enhanced if the water molecule is dissociated. Pure water is transparent to photons with energies up to about 6.9 eV (wavelengths of about 180 nm), but the absorption coefficient is about one million times larger at 12 eV [62]. The photon absorption in the VUV range is due to H–OH bond scission (water dissociation). The photons generated inside the cellulose samples upon electron-beam irradiation are, therefore, likely to provide the radicals needed to attack the β(1→4) bond. In fact, Henniges et al. [63] found more pronounced modifications in the case of wet cellulose samples.

The rather unexpected fact that all authors who have tackled cellulose depolymerization by electron-beam irradiation obtained results that fit the general curve (Figure 3 and Figure 4), irrespective of electron energy or sample thickness, is therefore explained by secondary effects, triggered by the primary electrons. The primary mechanism of cellulose depolymerization is the scission of the β(1→4) bond by particles (electrons and photons) with energies of around 10 eV. Less energetic particles (with energy below the threshold for bond scission) will only heat the sample (excite phonon vibrations). More energetic particles will predominantly cause ionization, i.e., the displacement of an electron with significant energy from the original binding site. In the case of energetic photons, the effect is called the “photo effect” if the electrons are displaced near the surface. A spectrum of such photoelectrons (together with the secondary electrons emitted by scattering of the photoelectrons) is shown in Figure 9.

The depolymerization efficiency should depend on the thickness of irradiated cellulose samples if the thickness is much smaller than the penetration depth (Figure 7). Unfortunately, the authors whose results are shown in Figure 3, Figure 4 and Figure 5 have not reported the thicknesses. Only Vasilieva et al. [41] mentioned “powdered cellulose”. If the powders were microscopic and the electrons were energetic (e.g., 10 MeV), most primary electrons and high-energy X-rays would miss the powders. However, Vasillieva used 30-keV electrons, so most of them did the job.

## 3. Conclusions

Based on the above considerations, the fast electrons used to treat cellulose serve as vectors for less-energetic particles formed within the cellulose samples. The crucial step in the depolymerization of samples thicker than the penetration depth of fast electrons is the electron energy loss via radiative mechanisms, i.e., the formation of energetic photons. The penetration depth of energetic photons is roughly 1000 to 10,000 times that of electrons, so 10 MeV electrons will generate energetic protons that penetrate the samples and stimulate reactions in a volume more than 10 cm deep within the cellulose samples. The energetic photons cause the formation of free (and less energetic) electrons in the bulk cellulose. The less energetic electrons produce even less energetic X-rays, which in turn trigger an avalanche of electrons with sufficient energy to drive bond scission deep inside the cellulose samples. Some electron energy is also spent on the formation of UV and VUV photons, which are also appropriate for the scission of bonds in cellulose.

Any attempt at commercializing a technology requires an estimate of its energy efficiency. Theoretically, the energy needed for scission of both the β(1→4) bond and the H–OH bond is roughly 10 eV. The number of the β(1→4) bonds in cellulose is roughly 10^24^ kg^−1^. So, the theoretically minimal energy dose for complete cellulose depolymerization is 10^25^ eV kg^−1^, or roughly 10^6^ J/kg (10^3^ kGy). Theoretically, complete cellulose depolymerization is feasible using standard electron-beam sources, but in practice, numerous effects make the required dose much larger. Namely, all particles below the threshold for bond scission will lose the energy to phonon excitation, i.e., heating. Furthermore, reversible effects, such as re-establishing the original bond, are likely to occur, and the particles will also break other bonds. Whatever the configuration, some energy will be lost to backscattered electrons and X-rays leaving the cellulose. Finally, it should be noticed that the intramolecular and intermolecular bonds play a significant role in any attempt to depolymerize cellulose. The synergy of all effects results in measured depolymerization rates that are much lower than theoretical prediction.

The electron beam sources have been optimized long ago. The energy is spent on heating the cathode to ensure large thermal emission and accelerating the electrons. The cathode and the acceleration unit should be placed in a high vacuum to avoid cathode degradation and electron scattering. It is impractical, if not impossible, to keep wet cellulose samples in high vacuum, but hermetically tight, semi-transparent windows are available nowadays and are used to separate high-vacuum chambers from liquids [64].

The experimental energy efficiency of depolymerization is much lower than the theoretical maximum. A depolymerization rate (i.e., the ratio of the initial to final DP) of 10 is achieved at an energy dose of roughly 70 kJ/kg. The specific heat capacity of cellulose typically ranges from 1.20 to 1.50 kJ/kg K. The samples exhibiting a depolymerization rate of 10 will experience a temperature rise of about 35 K. The heating is thus not negligible but still much less than the heating experienced when using thermal hydrolysis. In conclusion, electron-beam treatment is a useful method for partial depolymerization of cellulose. However, the treatment will be too energy- (and time-) demanding to replace other techniques for the production of sugars from cellulosic biomass.

## Data Availability

No new data were created or analyzed in this study.

## Abbreviations

The following abbreviations are used in this manuscript:

DP	Degree of polymerization
UV	Ultraviolet
VUV	Vacuum ultraviolet
